# Structural Disorder in Layered Hybrid Halide Perovskites: Types of Stacking Faults, Influence on Optical Properties and Their Suppression by Crystallization Engineering

**DOI:** 10.3390/nano11123333

**Published:** 2021-12-08

**Authors:** Andrei S. Tutantsev, Ekaterina I. Marchenko, Natalia N. Udalova, Sergey A. Fateev, Eugene A. Goodilin, Alexey B. Tarasov

**Affiliations:** 1 Laboratory of New Materials for Solar Energetics, Faculty of Materials Science, Lomonosov Moscow State University, 1 Lenin Hills, 119991 Moscow, Russia; tut.andrey.serg@gmail.com (A.S.T.); marchenko-ekaterina@bk.ru (E.I.M.); natalie.fnm@gmail.com (N.N.U.); saf1al@yandex.ru (S.A.F.); goodilin@yandex.ru (E.A.G.); 2 Department of Geology, Lomonosov Moscow State University, 1 Lenin Hills, 119991 Moscow, Russia; 3 Department of Chemistry, Lomonosov Moscow State University, 1 Lenin Hills, 119991 Moscow, Russia

**Keywords:** layered hybrid halide perovskites, low-dimensional crystal structure, stacking faults, defects, structural disorder, additives, crystallization engineering

## Abstract

Layered hybrid halide perovskites (LHHPs) are an emerging type of semiconductor with a set of unique optoelectronic properties. However, the solution processing of high-quality LHHPs films with desired optical properties and phase composition is a challenging task, possibly due to the structural disorder in the LHHP phase. Nevertheless, there is still a lack of experimental evidence and understanding of the nature of the structural disorder in LHHPs and its influence on the optical properties of the material. In the current work, using 2D perovskites (C_4_H_9_NH_3_)_2_(CH_3_NH_3_)_n−1_Pb_n_I_3n+1_ (further BA_2_MA_n−1_Pb_n_I_3n+1_) with n = 1–4 as a model system, we demonstrate that deviations in LHHPs optical properties and X-ray diffraction occur due to the presence of continuous defects—Stacking Faults (SFs). Upon analyzing the experimental data and modeled XRD patterns of a possible set of stacking faults (SFs) in the BA_2_MAPb_2_I_7_ phase, we uncover the most plausible type of SFs, featured by the thickness variation within one perovskite slab. We also demonstrate the successful suppression of SFs formation by simple addition of BAI excess into BA_2_MA_n−1_Pb_n_I_3n+1_ solutions.

## 1. Introduction

The family of layered hybrid halide perovskite-like compounds, often referred to as “two-dimensional (2D) hybrid halide perovskites”, is a new class of materials demonstrating a set of unique functional properties, such as record-breaking quantum yield of photo- and electroluminescence, tunable narrow or broad white emission, and superior exploitation stability comparing to the APbX_3_ halide perovskites [1]. The general formula for the most common structural type of LHHPs is (A’)_2/q_A_n−1_Pb_n_X_3n+1_ where [A’]^q+^ is a single or double charged bulky organic cation in the interlayer space, and A^+^ is a small cation (e.g., CH_3_NH_3_^+^ = MA^+^) stabilizing [A_n−1_Pb_n_X_3n+1_]^2/q−^ slab built from the n layers of corner-sharing [PbX_6_]^4−^ octahedra [2,3,4]. The (A’)_2/q_A_n−1_Pb_n_X_3n+1_ perovskites form a homologous series with optoelectronic properties monotonically changing with the n number [5]. Owing to structural versatility and outstanding optoelectronic properties [6], LHHPs are considered perspective materials for use in perovskite solar cells [7,8,9,10], light-emitting diodes (LED) [11,12,13,14], lasers [15,16], field-effect transistors [17,18], photodetectors [19,20], etc.

Unfortunately, it is hard to obtain phase-pure LHHPs polycrystalline thin films with a target n number via solution processing [21,22] that sophisticates the control of optical properties of the material. A typical LHHPs film demonstrates heterogeneity in optical properties manifested as coexistence of multiple absorption/emission bands corresponding to the phases with different n—only one of which is associated with the target LHHP phase. However, the X-ray diffraction (XRD) data in the abovementioned articles are not informative enough to support the presence of phase impurities in the films. XRD patterns usually demonstrate only two reflexes from (111) and (222) crystallographic planes positioned around 14° and 28° 2θ, respectively [22]. Such a discrepancy between optical spectroscopy and X-ray diffraction data could originate from the presence of defects and disorder in LHHPs polycrystalline films. Pioneer experimental evidences of the disorder in BA_2_MA_n−1_Pb_n_I_3n+1_ (n = 3, 4) films were reported by Venkatesan et al. [23] and Tan et al. [24]. In these articles, the authors suggested the presence of stacking faults (SFs) in LHHPs films. However, this type of defect and its influence on the optical properties remains poorly investigated, highlighting the need for detailed research in this field to overcome the problem of defect formation and non-reproducibility of optical and functional properties of such materials.

In the present work, we thoroughly analyzed the XRD and optical spectroscopy data of solution-processed BA_2_MA_n−1_Pb_n_I_3n+1_ thin films with various n = 1−4, identifying the characteristic features of the structural disorder observed in this class of materials. We proposed a number of possible extended defect types in the LHHPs crystal structure and revealed important correlations between the defect type, XRD patterns, and optical properties. As a result, we suggest that the most plausible type of extended defects for the family of layered perovskites are the stacking faults, with thickness variation within one slab having a 1/2 octahedra shift. We also suggest a strategy to control the crystallization process of LHHPs films using an excess of BAI added to the solution, allowing us to obtain LHHPs films with improved crystallinity and optical properties.

## 2. Materials and Methods

Chemicals: Methylammonium iodide (CH_3_NH_3_I = MAI, 99%, Dyesol), butylammonium iodide (C_4_H_9_NH_3_I = BAI, GreatCell, Queanbeyan, Australia), lead iodide (PbI_2_, >98%, TCI, Tokyo, Japan), N,N-dimethylformamide (HCON(CH_3_)_2_ = DMF, anhydrous, 99.8%, Sigma-Aldrich, Darmstadt, Germany), and fluorine-doped tin oxide (FTO, ~7 Ω/sq, Sigma Aldrich) were commercially purchased.

Synthesis of perovskite films: (BA)_2_(MA)_n−1_Pb_n_I_3n+1_ films with n = 1–4 were prepared by spin-coating of stoichiometric solutions of BAI, MAI, and PbI_2_ in DMF with a fixed [Pb^2+^] concentration of 0.7 M. For some samples, excess BAI was added during the solution preparation. Before film deposition, FTO or glass substrates were sequentially cleaned by Hellmanex III (2% aqueous solution), isopropyl alcohol, and distilled water within an ultrasonic bath (10 min each). Finally, UV ozone treatment was provided for 15 min by using Ossila UV Ozone Cleaner (Sheffield, England). Spin-coating was performed in a glove box with dry air atmosphere at 25 °C. An amount of 20 μL of perovskite solution was spread on the substrates before spinning, and then the substrates were accelerated to rotate at 6000 rpm for 2 s and spun for 20 s. After that, the films were annealed at 100 °C for 10 min.

Solubility measurements: Solubility of BA_2_MA_n−1_Pb_n_I_3n−1_ with n = 1–4 and MAPbI_3_ perovskites in DMF was provided at 25 °C with the use of mixed stoichiometric BAI, MAI, and PbI_2_ precursor powders in a dry glove box atmosphere.

X-ray powder diffraction: The XRD analysis was performed using a Bruker Advance D8 diffractometer in Bragg–Brentano geometry with CuK_α_ radiation (λ = 1.5418 Å). XRD patterns were recorded in the 3°–35° 2θ range with 0.1 s per dot and 0.02° step.

Absorption and photoluminescence: The steady-state photoluminescence (PL) measurements were performed using a home-built microscope with a Flame VIS-NIR CCD spectrometer (Ocean Optics, Orlando, FL, USA). The samples were photoexcited using a 405 nm laser diode operating in CW mode. The absorption spectra were recorded in the transmission mode on a Perkin Elmer Lambda 35 spectrophotometer (Waltham, MA, USA) in the wavelength range of 350–850 nm.

X-ray Diffraction Simulation: Powder diffraction patterns were simulated using Mercury software [25] with a source wavelength λ = 1.54056 Å (corresponding to CuK_α1_ radiation). Supercells were composed of 5 × 1 × 25 unit cells with 50 stacking layers, 4500 atoms for BA_2_MAPb_2_I_7_, 5 × 1 × 25 unit cells with 50 stacking layers, and 6500 atoms for BA_2_MA_2_Pb_3_I_10_. For all the atoms, isotropic atomic displacements of 0.05 Å were assigned. Peaks were assumed to be symmetric with a pseudo-Voight shape and 0.1° full width at half maximum (according to the experimental data). The VESTA program [26] was used to visualize the crystal structures.

Only a random disorder of the structures has been modeled. The c (b) lattice parameter of a matrix structure has been considered to be a prescribed value that was taken to be equal to the value known for the defect-free structure. The thickness of a new structural fragment arising from the presence of the SF was also preset using the geometrical parameters of structural fragments of experimentally investigated structures. The assumption of a constant value of the c lattice parameter should be taken with caution because various authors report different c (b) values for the same material [27]. There are strong grounds for believing that the thickness of the new structural fragments may deviate significantly from the values determined from the reported c (b) lattice parameters.

## 3. Results and Discussion

**Figure 1:**
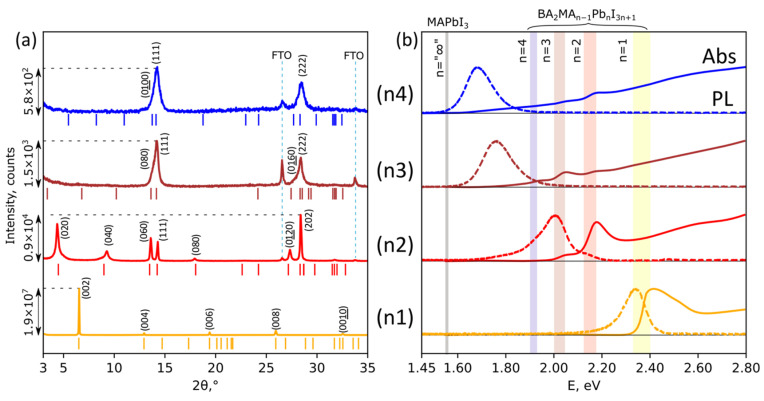
Experimental XRD patterns (**a**), optical absorption (solid lines) and photoluminescence (dashed lines) spectra (**b**) of BA_2_MA_n−1_Pb_n_I_3n+1_ films with n = 1–4. Vertical lines in (**a**) correspond to theoretical XRD peak positions for each phase; colored vertical bars in (**b**) represent positions of exciton emission (left border) and exciton absorption peaks (right border) of pure BA_2_MA_n−1_Pb_n_I_3n+1_ phases with n = 1–4.

The BA_2_MA_n−1_Pb_n_I_3n+1_ thin films with n = 1–4 were prepared by spin-coating of DMF solutions and characterized by XRD, optical absorption, and PL spectroscopy. Hereinafter, we refer to BA_2_PbI_4_ as n1, BA_2_MAPb_2_I_7_ as n2, BA_2_MA_2_Pb_3_I_10_ as n3, and BA_2_MA_3_Pb_4_I_13_ as n4. According to experimental results, n1 films were reproducibly phase-pure and were characterized by intensive (0k0) reflexes in XRD patterns, and also by single exciton absorption and PL peaks of the target BA_2_PbI_4_ phase [5] (Figure 1, (n1)). However, for n2 samples, we observed a noticeable structural disorder characterized by the three orders of magnitude decrease in diffraction intensity, and by counter-directional shifts and asymmetrical widening of (0k0) series of XRD reflexes with no signs of impurity phases (Figure 1a, (n2)). On the contrary, optical spectroscopy data show the presence of higher-n impurities in the n2 film: (i) the absorption spectrum includes mostly the n2 phase with the n3 phase sign; (ii) the PL spectrum consists of an asymmetrical emission line with the maximum at 2 eV corresponding to the n3 phase and also less intensive PL of n2, n4, and even n > 4 LHHPs (Figure 1b, (n2)). In the case of n3 and n4 films, we observed further decrease in diffraction intensity and the disappearance of the majority of (0k0) XRD reflexes, with only two widened peaks left at 14° and 28° 2θ corresponding to (111) and (222) crystallographic planes (Figure 1a, (n3, n4)). Absorption spectra of n3 and n4 films consisted of many components, starting from n2 and up to n ~ 7 (Figure 1b, (n3, n4)). PL emission of these samples had a maximum of 1.76 eV for n3 and at 1.68 eV for n4, which corresponds to higher-n members of the BA_2_MA_n−1_Pb_n_I_3n+1_ family.

On the one hand, all optical spectroscopy observations can be attributed to the presence of lower- and/or higher-n impurities in the films of LHHPs with n ≥ 2 (Figure 1b). On the other hand, XRD data reveal no presence of any phase impurities in all LHHP samples, which means that the common concept of 2D perovskite film with multiple phases [21,22] is not applicable in our case (Figure 1a). Such a discrepancy between XRD and optical spectroscopy data requires a deeper analysis of the obtained experimental results to reveal its origin.

**Figure 2:**
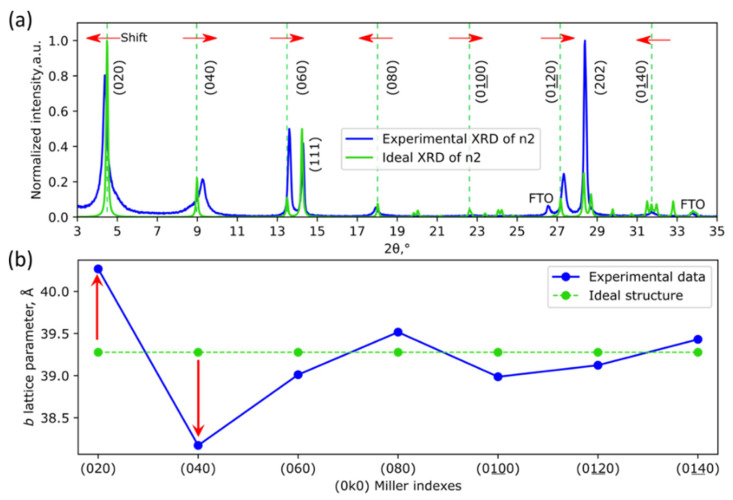
(**a**) Experimental (blue) and ideal (green) XRD patterns of BA_2_MAPb_2_I_7_ film. Green vertical dashed lines correspond to theoretical positions of (0k0) reflexes. Red arrows indicate the directions of each peak shift. (**b**) Experimental (blue) and theoretical (green) values of the lattice parameter *b* calculated from positions of each (0k0) reflex in the corresponding XRD pattern in (**a**). Red arrows illustrate the counter-directional change of the cell parameter b.

The detailed analysis of the n2 XRD pattern reveals two unusual features: asymmetrical broadening and a counter-directional shift of (0k0) series reflexes (Figure 2a). The (0k0) reflexes correspond to the direction of layer stacking. The counter-directional shift of (0k0) reflexes cannot be explained by the presence of the mixture of any known n2 polymorphs. The dependency of the cell parameter b from the value of the Miller index k is found to be irregular (Figure 2b). Such an irregular change in the cell parameter, along with asymmetrical broadening, is one of the typical features of stacking faults (SFs) in various layered compounds [28]. For example, in layered superconductive oxides with the closest packing, SFs are presented as the alternations of two types of structural fragments. However, with non-close packed layered hybrid perovskites, stacking faults should have a completely different nature.

For LHHPs, the general principle of all possible stacking faults is the deviation of the slab thickness n_i_ in the real structure from the ideal case. Such a deviation can occur due to the alternation of normal slabs with different thicknesses (further type “SF-A”) or due to a thickness variation within one slab (further type “SF-B”).

**Figure 3:**
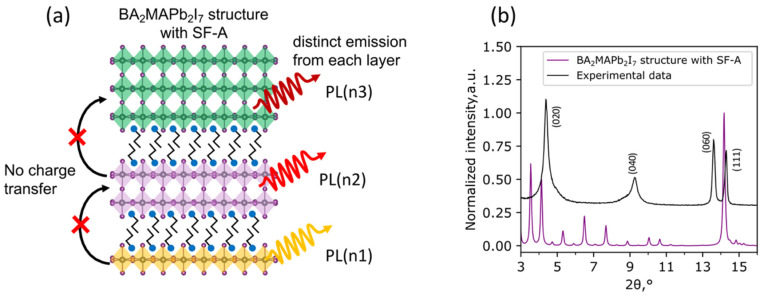
(**a**) Schematic model structure of n2 LHHP with type-A stacking faults. Curved lines illustrate separate emissions from each isolated slab with different n. (**b**) Comparison of calculated XRD pattern from the n2 structure distorted by SF-A with experimental XRD pattern from n2 thin film.

The stacking faults with type A, in the case of n2 layered perovskite, are represented as a combination of normal n = 2 layers with some amount of n = 1 and n = 3 substituting slabs, ultimately preserving the nominal stoichiometry (BA_2_MAPb_2_I_7_) of the material (Figure 3a). However, optical properties of such a material should demonstrate the presence of all components with different thickness (in the case of n2, we expect to observe PL and absorption lines from n1, n2, and n3 slabs) due to the absence of a charge transfer between slabs isolated from each other by organic interlayer cations (Figure 3a). Moreover, the simulated XRD pattern of n2 with SF-A, consisting of a large number of reflexes from each component, matches no experimental data from the n2 thin film (Figure 3b). Additionally, the XRD pattern of the scraped off LHHP film contains no intense peaks from the (0k0) planes, as well as different n components (Figure S1), supporting the abovementioned observations and, thus, assuming the irrelevance of type-A stacking faults for the layered hybrid halide perovskites.

**Figure 4:**
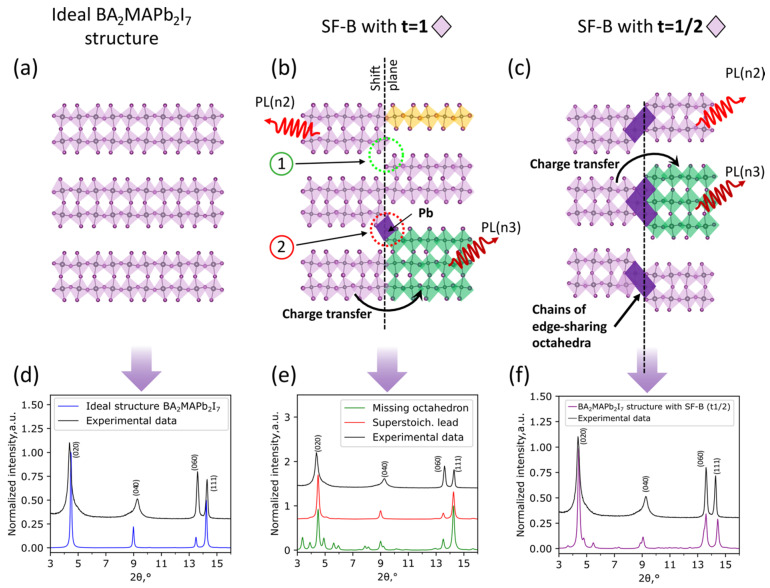
(**a**–**c**) Schemes of n2 crystal structure with: (**a**) the absence of structural disorder; (**b**) SF-B(t1) defects with n1 and n3 substituting fragments, and with either unoccupied\missing octahedra (1) or additional lead cations forming edge-sharing “bridge” octahedra (2) between neighboring slabs; (**c**) SF-B(t^1/2^) defects with n3 substituting fragments and edge-sharing octahedra on the shift line. Curved arrows illustrate preferred PL emission from a narrower bandgap (higher n) fragments of perovskite slab. (**d**–**f**) The comparison between experimental (black) and calculated XRD patterns of each type of defective n2 structure types illustrated above. The organic cations are not shown for clarity.

The second possible type of SFs with thickness variation within one slab (SF-B) is primarily characterized by a shift of [PbI_6_] octahedra inside each distorted perovskite slab along the stacking direction (b-axis). The value of shift is characterized by translation vector [29] (t), which can be equal to 1 (whole [PbI_6_] octahedron thickness, Figure 4b) or equal to 1/2 (a half of octahedron thickness, Figure 4c). Hereafter, we will denote these two kinds of stacking faults as SF-B(t1) and SF-B(t^1/2^). Other shift values are much less possible in this system due to the absence of mutual crystallographic planes in a distorted perovskite structure. To elucidate the most probable type of SF-B, we calculated and compared XRD patterns for the BA_2_MAPb_2_I_7_ phase without defects [27] (Figure 4d) and with three possible scenarios of SF-B defects formation (Figure 4e,f), as discussed in detail further.

The SF-B(t1) defect type with an octahedron shift value of t = 1 is characterized by the presence of the shift plane on the border of SFs (Figure 4b). This border may contain two types of connection between neighboring slabs: (1) with unoccupied\missing octahedra or (2) with additional lead cations forming edge-sharing “bridge” octahedra (Figure 4b). In contrast to the SF-A type, charge carriers are able to transfer across the shift plane from different fragments of perovskite slabs in SF-B, leading to the predominant PL emission from lower-bandgap (higher n) structural fragments. Interestingly, the calculated XRD patterns of the n2 perovskite with SF-B(t1), with missing octahedra, contain a lot of additional reflexes around (020) and (040) peaks (Figure 4e, green), while the presence of additional Pb^2+^ cations leads to the minor change of the XRD pattern with respect to the ideal n2 crystal structure (Figure 4e, red). However, this stacking fault type does not demonstrate an agreement with the experimental diffraction pattern for n2 LHHP, even with a high defect concentration (Figure S2). In view of this, we conclude that the SF-B(t1) defect type does not dominate in BA_2_MAPb_2_I_7_ polycrystalline films. The SF-B(t^1/2^) type is characterized by the shift value t = 1/2 and the presence of edge-sharing octahedra on the shift plane (colored purple in Figure 4c).

We also expect a strong decrease in XRD intensity and counter-directional irregular shift of the reflections for crystallographic planes primarily aligned along the perovskite slabs in the structure (such as 0k0 or hkl with high k/h and k/l ratio). The primary reason for these facts is an appearance of a new structural block with interlayer distances different from such an undistorted structure [28], which is a special case of SF-B with t = 1/2. At the same time, the (111) crystallographic planes of the layered structure, which contain the closest packed fragments of perovskite slabs, are expected to be the least prone to disorder and the consequent loss of intensity, which agrees well with experimental XRD patterns (Figure 2a).

With regard to optical properties of the n2 layered perovskite with SF-B(t^1/2^), we also expect a charge carrier transfer from wider to narrower bandgap perovskite fragments, allowing a preferred PL emission from structural blocks with higher n (Figure 4c), as is usually observed for other layered perovskites [30,31]. The presence of wider bandgap edge-sharing octahedra could play a role in energetic barriers, but we suppose the small thickness of such PbI_2_-like fragments allows electrons to tunnel through them. An analysis of calculated XRD patterns for n2 LHHP with SF-B(t^1/2^) type of defects reveals an asymmetric broadening and counter-directional shift of (020), (040), and (060) reflexes similar to the experimental XRD pattern of the n2 film (Figure 4f). Along with XRD, optical properties of such LHHP with the SF-B(t^1/2^) defects would correlate well with experimental optical properties of the n2 film (superposition of n3 + n2 + n4 emission and n2 + n3 absorption bands, Figure 1b) due to the abovementioned charge transfer from wider to narrower bandgap regions. A combination of these agreements supports the assumption of SF-B(t^1/2^) dominance as the main defect type in BA_2_MAPb_2_I_7_ films and analogous phases with other n numbers.

It is interesting to note that the structures with SF-B(t^1/2^) cannot preserve their initial stoichiometry due to the presence of edge-sharing octahedra with “PbI_2_” stoichiometry on the shift plane and variable proportions of different (n1, n2, n3, etc.) fragments. Thus, to keep BA_2_MA_n−1_Pb_n_I_3n+1_ stoichiometry of the original solution in the resulting films of LHHPs with SF-B(t^1/2^) containing PbI_2_-like fragments, the prepared material should contain amorphous impurities of BAI.

**Figure 5:**
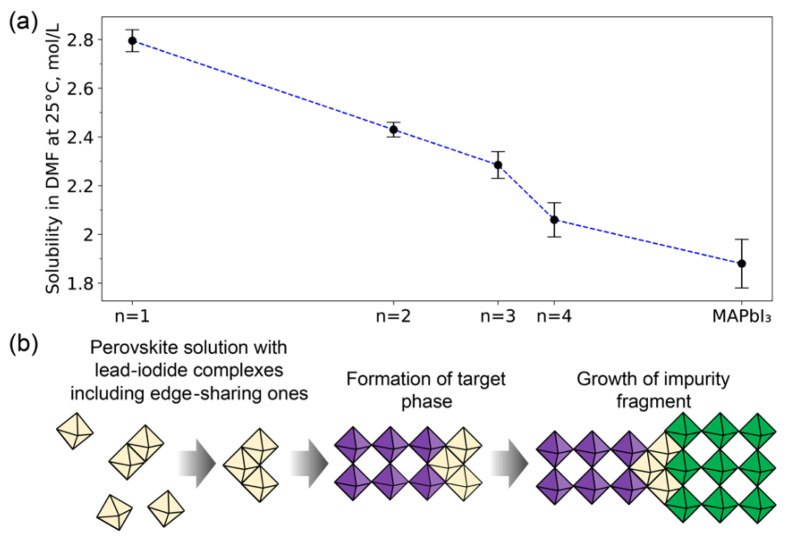
(**a**) Solubility of BA_2_MA_n−1_Pb_n_I_3n−1_ with n = 1–4 and MAPbI_3_ in DMF at 25 °C. (**b**) Schematic illustration of layered perovskite crystallization with ongoing formation of type B stacking faults with octahedra shift t = 1/2.

The abovementioned features of the structural disorder in LHHPs films are supposed to originate from the crystallization mechanism of layered perovskites from solutions. The process of perovskite film crystallization is governed by rapid nucleation due to rather high supersaturation. In the family of 2D perovskites, different members can have close solubility, thus ultimately leading to the formation of impurities with n ≠ n_i_. We measured the solubilities (S) of n1, n2, n3, and n4 LHHPs and MAPbI_3_ in DMF (Figure 5a), and we found it decreasing monotonically from n1 with S ~ 2.8 M to MAPbI_3_ with S ~ 1.9 M. At the same time, the difference between the solubilities of n2 and n3 is rather small, which may explain simultaneous formation of n2 and n3 fragments. However, the solubility of n1 perovskite is much higher than solubilities of n2 and n3 members, hindering crystallization of the BA_2_PbI_4_ phase. Instead, additional BAI and PbI_2_ compounds could form amorphous phases and structural inclusions, respectively. The presence of PbI_2_-like inclusions in type-B stacking faults, theoretically predicted above, could be derived from the structure of lead iodide complexes in perovskite solution. It is known that DMF solvent tends to form coordination complexes with PbI_2_, where PbI_6_ octahedra are connected predominantly by edges or faces, leading to the formation of polynuclear complexes (the “building blocks”) [32,33]. Incorporation of such building blocks into the LHHPs crystal structure during thin film crystallization may promote the formation of stacking faults with a shift value of t = 1/2 (Figure 5b).

**Figure 6:**
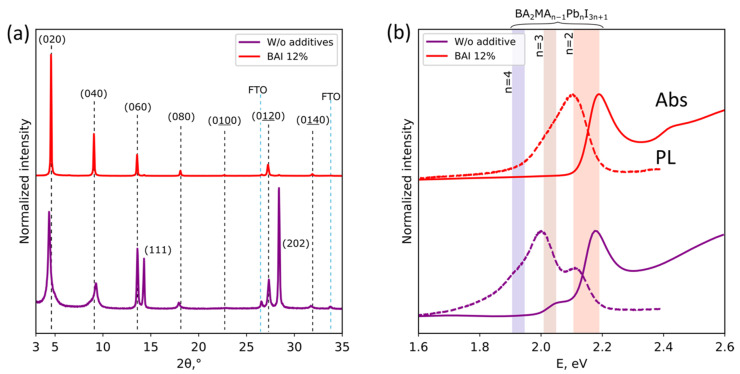
(**a**) XRD patterns and (**b**) UV–vis absorption (solid line) and PL (dashed line) spectra of BA_2_MAPb_2_I_7_ films without additives (violet) and with addition of 12% excess BAI (red).

To minimize the amount of stacking faults in layered perovskite thin films, we suggest using an excess of the BAI component in the perovskite solution. The excess of I^−^ ions in the solution would prevent the formation of polynuclear species [Pb_x_I_2x+n_]^n−^ in the solution [32] and may ultimately decrease the probability of SFs formation. In turn, the excess of BA^+^ shifts the solubility equilibrium between the phases with different n and the solution in favor of lower-n LHHPs formation. The latter is caused by the principle of Le Chatelier: increasing the concentration of [BA^+^] leads to the decrease in solubility of LHHPs with a higher content of butylammonium. For the n2 perovskite, the optimal excess of BAI was found to be about 12% since the XRD pattern (Figure 6a) and optical properties (Figure 6b) became closer to the defect-free BA_2_MAPb_2_I_7_. A further increase in BAI concentration led to the formation of an undesired BA_2_PbI_4_ impurity phase (Figure S3). It was also shown that BAI addition did not affect the long-term thermal stability of n2 perovskite (Figure S4).

However, this approach demonstrates only partial effectiveness in the case of higher-n LHHPs due to the diminishing solubility difference between neighboring members of BA_2_MA_n−1_Pb_n_I_3n+1_. For example, addition of excess BAI into the BA_2_MA_2_Pb_3_I_10_ solution increased the diffraction intensity, especially (0k0) series reflexes, with simultaneous narrowing of each reflex (Figure S5). However, optical properties of the n3 film with a “phase-pure” XRD pattern still demonstrate heterogeneity, revealing some structural disorder or nanoscale impurities (Figure S6). Therefore, the use of excess iodide of bulky organic cation in LHHPs solution allows one to control the optical properties of 2D perovskite thin films only partially.

## 4. Conclusions

To sum up, we demonstrated that the thin films of BA_2_MA_n−1_Pb_n_I_3n+1_ layered perovskites with n ≥ 2 tend to crystallize with a pronounced structural disorder. The most probable type of extended defect in such systems is suggested to be stacking faults within perovskite slabs, consisting of structural blocks with different numbers of [PbI_6_] octahedra layers (n_i_ ± 1, ± 2, etc.), which are connected to each other via edge-sharing octahedra with a shift value t = 1/2, equal to the half-length of the [PbI_6_] octahedron. This SFs type is characterized by the asymmetrical broadening and counter-directional shifts of XRD reflexes, as well as by optical absorption and emission of the structural blocks with different n. This agrees well with experimental data, in contrast to all other assumable types of SFs. We assume that the formation of such special types of stacking faults originates from the mechanism of perovskite crystallization from solution in highly coordinating solvents. Firstly, it was found that the solubility difference between neighboring BA_2_MA_n−1_Pb_n_I_3n+1_ perovskites strongly decreases with n, becoming small already between the n2 and n3 LHHPs members, and leads to simultaneous formation of 2D perovskites with different n. In addition, the presence of edge-sharing polynuclear Pb-I complexes in perovskite solutions may play the role of connecting sites between perovskite structural blocks with different thickness n. To suppress these factors and to obtain phase-pure and highly crystalline LHHPs films, we successfully applied the admixing of excess BAI into the perovskite solution. This approach allows one to increase the solubility of polynuclear lead iodide complexes and promotes the formation of LHHP phases with a target n number. Therefore, the use of various additives into layered perovskites solution seems to be a promising strategy for obtaining phase-pure LHHPs films with a low level of structural disorder.

## Data Availability

Not applicable.

## Supplementary Materials

The following are available online at https://www.mdpi.com/article/10.3390/nano11123333/s1, Figure S1: Comparison of XRD patterns of n3 film and scraped off powder from n3 film; Figure S2: Calculated XRD patterns of BA_2_MAPb_2_I_7_ structure with different SF-B(t1) defect concentrations in the case of additional lead cations (a) and unoccupied\missing octahedra (b) on the shift plane; Figure S3: XRD patterns of BA_2_MAPb_2_I_7_ films with different concentrations of BAI excess (0–25%) given in percentage of [Pb^2+^] concentration in solution; Figure S4: XRD patterns of BA_2_MA_2_Pb_3_I_10_ films with different concentrations of BAI excess given in percentage of [Pb^2+^] concentration in solution; Figure S5: UV-Vis absorption (a) and PL (b) spectra of BA_2_MA_2_Pb_3_I_10_ films with different concentrations of super stoichiometric BAI given in percentage of [Pb^2+^] concentration in solution. Figure S6: UV-vis absorption (a) and PL (b) spectra of BA_2_MA_2_Pb_3_I_10_ films with different concentrations of super stoichiometric BAI given in percentage of [Pb^Fi+^] concentration in solution.
