# Supplementary material for: Structural Disorder in Layered Hybrid Halide Perovskites: Types of Stacking Faults, Influence on Optical Properties and Their Suppression by Crystallization Engineering
Source: Nanomaterials (Basel). 2021 Dec 8;11(12):3333. doi: 10.3390/nano11123333 (PMC8703331; doi:10.3390/nano11123333)
Supplement: Supplementary file 1 [file nanomaterials-11-03333-s001.zip › nanomaterials-1490110-supplementary.pdf]

supplementary materials

Structural Disorder in Layered Hybrid Halide Perovskites: Types of Stacking Faults, Influence on Optical Properties and Their Suppression by Crystallization Engineering

Andrei S. Tutantsev [1,†], Ekaterina I. Marchenko [1,2,†], Natalia N. Udalova [1], Sergey A. Fateev [1], Eugene A. Goodilin [1,3] and Alexey B. Tarasov [1,3,*]

[1] Laboratory of New Materials for Solar Energetics, Faculty of Materials Science, Lomonosov Moscow State University, 1 Lenin Hills, 119991 Moscow, Russia; tut.andrey.serg@gmail.com (A.S.T.); marchenko-ekaterina@bk.ru (E.I.M.); natalie.fnm@gmail.com (N.N.U.); saf1al@yandex.ru (S.A.F.); goodilin@yandex.ru (E.A.G.)

[2] Department of Geology, Lomonosov Moscow State University, 1 Lenin Hills, 119991 Moscow, Russia

[3] Department of Chemistry, Lomonosov Moscow State University, 1 Lenin Hills, 119991 Moscow, Russia

* Correspondence: alexey.bor.tarasov@yandex.ru

† These authors contributed equally to this work.

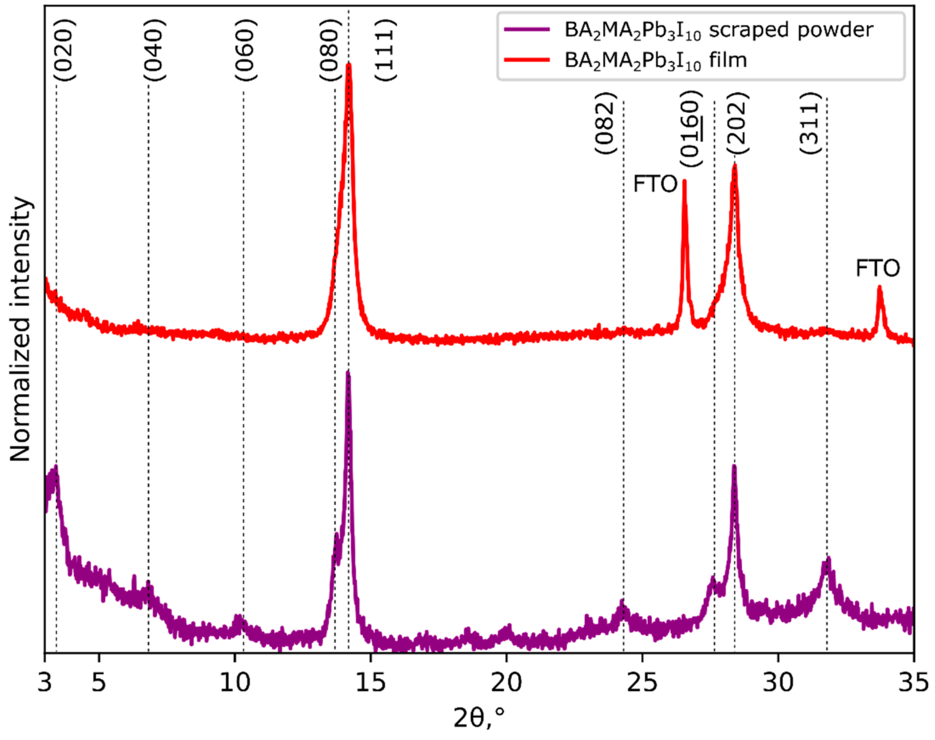

Figure S1. Comparison of XRD patterns of n3 film (red line) and scraped off powder from n3 film (purple line).

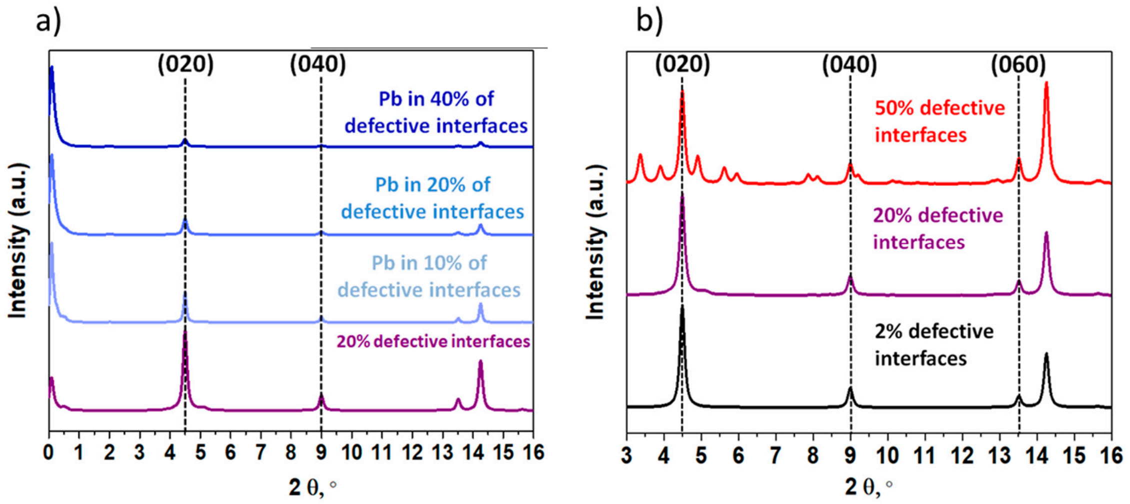

Figure S2. Calculated XRD patterns of $BA_2MAPb_2I_7$ structure with different SF-B(t1) defect concentrations in the case of additional lead cations (a) and unoccupied / missing octahedra (b) on the shift plane.

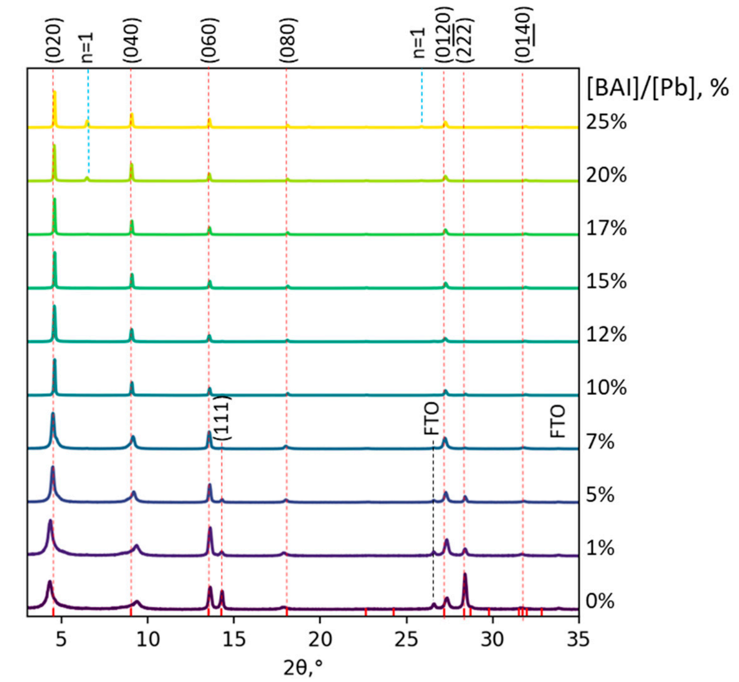

Figure S3. XRD patterns of $BA_2MAPb_2I_7$ films with different concentrations of BAI excess (0 – 25%) given in percentage of $[Pb^{2+}]$ concentration in solution.

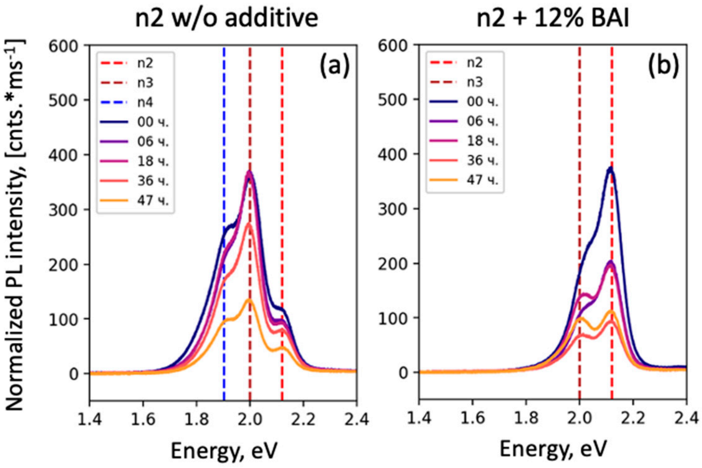

Figure S4. PL spectra of n2 perovskite without (a) and with 12% BAI additive (b) at different time of 100°C heating in inert dry atmosphere.

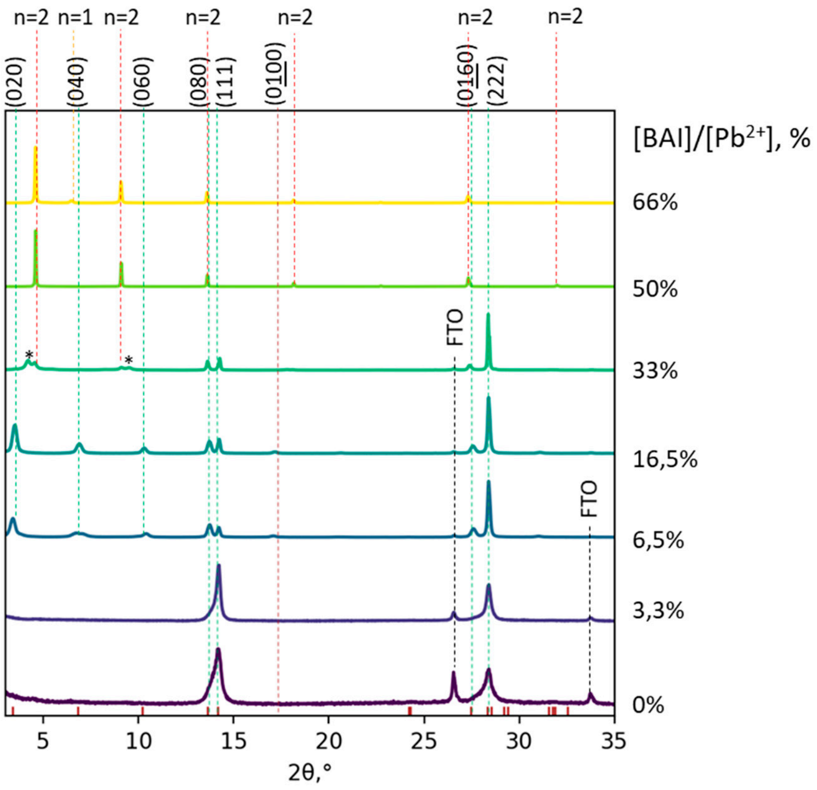

Figure S5. XRD patterns of $BA_2MA_2Pb_3I_{10}$ films with different concentrations of BAI excess given in percentage of [Pb^{2+}] concentration in solution.

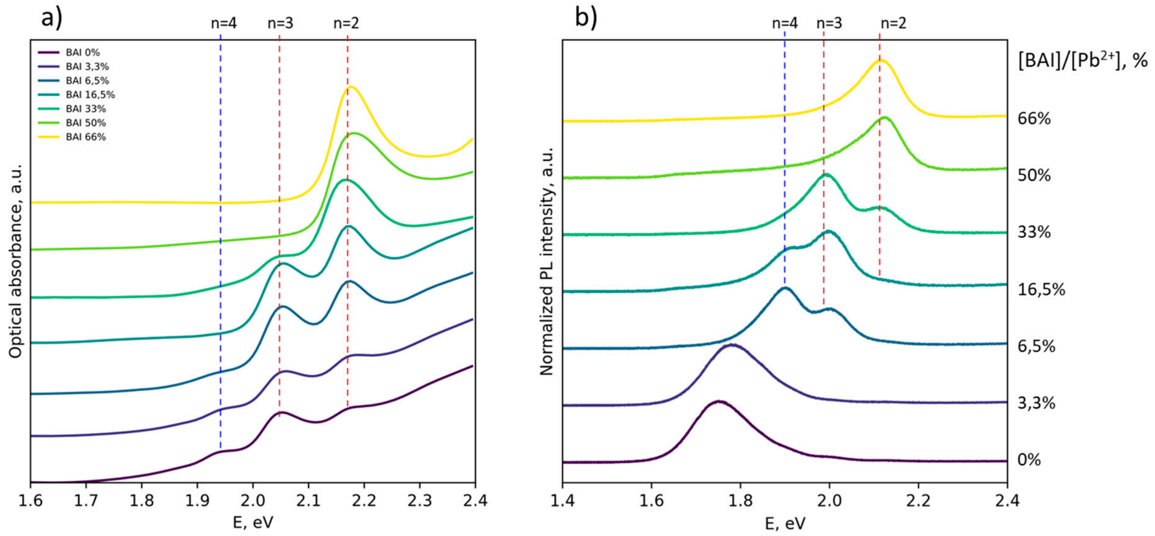

Figure S6. UV-vis absorption (a) and PL (b) spectra of $BA_2MA_2Pb_3I_{10}$ films with different concentrations of super stoichiometric BAI given in percentage of $[Pb^{2+}]$ concentration in solution.
